# A pilot study of a mindfulness informed yoga intervention in young people with psychosis

**DOI:** 10.1111/eip.13264

**Published:** 2022-02-01

**Authors:** Destiny O'Dea, Jackie Curtis, Alana Scully, Julia Lappin

**Affiliations:** ^1^ Faculty of Medicine University of New South Wales Kensington New South Wales Australia; ^2^ Early Psychosis Program, Bondi Junction Community Mental Health Centre South Eastern Sydney Local Health District New South Wales Australia

**Keywords:** adolescents, mental health, mindfulness, psychotic disorders, yoga

## Abstract

**Aim:**

To determine the acceptability and safety of a mindfulness informed yoga intervention as adjunct to usual care for young people with early psychosis.

**Methods:**

People aged 16–25 years attending a community‐based specialist early psychosis clinic were invited to participate in a 12‐week yoga intervention. The intervention consisted of 1‐h weekly classes of mindfulness informed yoga. Acceptability was measured by uptake, attendance and participants' satisfaction. Safety was measured by incidence of physical injury, participants' level of comfort, distress and anxiety during the sessions, and the following mental health outcomes: positive and negative psychotic, depression, anxiety and stress symptoms, sleep quality and functioning.

**Results:**

Of those who consented to the study, 80% (12) participated and on average attended 4.4 yoga classes. There were no physical injuries and participants reported minimal distress and anxiety. Post‐intervention, there was a significant reduction in anxiety symptoms and an improvement in function.

**Conclusions:**

Mindfulness‐based yoga interventions are both acceptable and safe as an intervention for youth with early psychosis. Though numbers were small, the study shows promise for yoga as a potentially useful intervention. Importantly, there was no deterioration in mental health outcomes. A larger trial evaluating clinical effectiveness is now timely.

## 1 INTRODUCTION

First episode psychosis (FEP) is a serious mental illness characterized by positive and negative psychotic symptoms and impaired cognitive function. Recovery based FEP services aim to embrace a holistic model of healthcare which addresses mental health symptoms, functional impairment and physical health inequity. Consequently, there is interest in the evaluation of yoga and mindfulness interventions to complement existing pharmacological and psychosocial treatments in improving outcomes in psychosis (Bird et al., 2010; Cella et al., 2017).

To date, there is limited evidence from a number of small studies that yoga or mindfulness interventions improve a range of patient outcomes in adults with schizophrenia. A small pilot randomized controlled trial (RCT) and a large RCT found that yoga improved positive and negative psychotic symptoms (Behere et al., 2011; Visceglia & Lewis, 2011). In contrast, another RCT found that yoga improved negative but not positive symptoms (Duraiswamy et al., 2007). Two RCT's found that yoga or exercise interventions improved socio‐occupational function (Behere et al., 2011; Duraiswamy et al., 2007). A further RCT demonstrated that interventions combining yoga and physical exercise improved cognitive function (Bhatia et al., 2017). Finally, two RCT's found that yoga improved quality of life (Duraiswamy et al., 2007; Visceglia & Lewis, 2011). The studies to date are limited by poor study design including sample size and selection, being single‐blinded and having minimal longitudinal follow‐up.

Fewer studies have assessed the clinical effectiveness of yoga in FEP populations. An RCT compared a 12‐week yoga and aerobic exercise program to determine its impact on neurocognitive function in 124 adult women aged 16–60 years with early psychosis (Lin et al., 2015). Both yoga and aerobic exercise groups showed improved working memory, positive and negative symptoms and depressive symptoms. The yoga group additionally showed improvements in verbal acquisition and attention. Importantly, this study showed that these improvements were maintained at 18‐month follow‐up. Participants who had more severe symptoms of psychosis at baseline were more likely to be non‐adherent to the intervention (Lin et al., 2015). While these findings are promising, the study excluded participants with secondary substance abuse disorder. A more heterogeneous cohort is needed to clarify the extent to which the findings may be generalized, especially given the high prevalence of substance use disorders in FEP populations (Visceglia & Lewis, 2011). A non‐randomized controlled pilot study of 33 people aged 15–25 years with FEP showed multi‐modal lifestyle interventions including yoga, mindfulness, nutritional education and group discussion are feasible and improve mental health outcomes (Usher et al., 2018). However, caution must be taken when interpreting their findings due to the small sample size and pragmatic nature of the study. A systematic review found that group delivery of mindfulness interventions in people with psychosis reduced depression symptoms when compared to individual delivery (Louise et al., 2018). While individuals with FEP were included in this sample, the majority had schizophrenia. A significant limitation of this systematic review is the heterogeneity and quality of evidence of the original studies. Importantly, no study has included ultrahigh risk for psychosis (UHR) populations. The current pilot study aimed to evaluate the acceptability and safety of a mindfulness informed yoga intervention (MIYI) in young people with FEP or UHR. To our knowledge, no previous studies have investigated acceptability and safety of yoga in young people with FEP or UHR. It was hypothesised that MIYI would be deemed acceptable and safe within the target population.

## 2 METHODS

### 2.1 Design and setting

This pilot study utilized a single‐arm, pre‐post, 12‐week uncontrolled design. Ethics approval was received from the South Eastern Sydney Local Health District Human Research Committee (Reference 17/298; LNR/17/POWH/580). The study was conducted between May and September 2019 at the Bondi Junction Community Mental Health Centre (BJCMHC).

### 2.2 Participants

**FIGURE 1:**
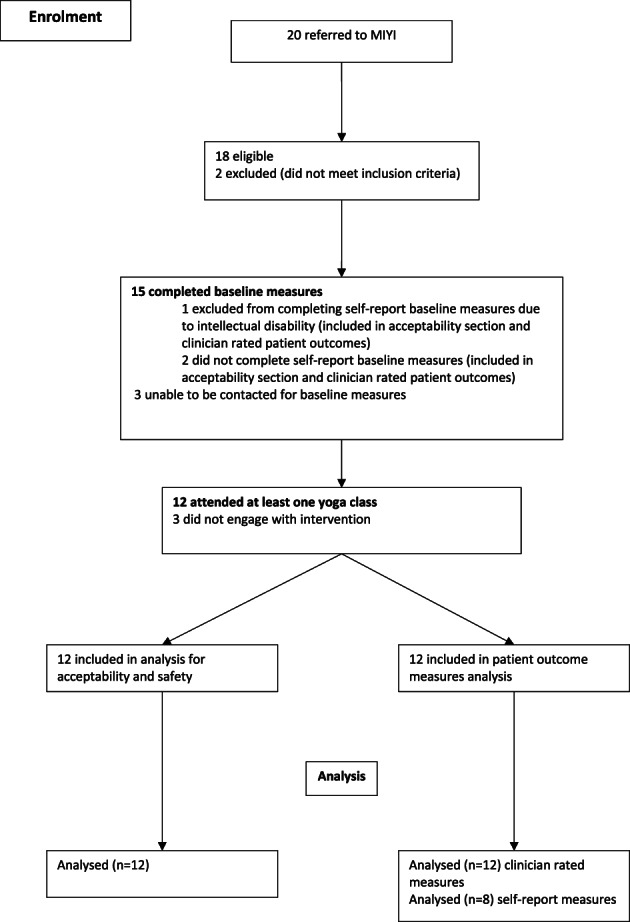
Flowchart of enrolment and study process—explains the process of enrolment, uptake of intervention and number of participants included in the analysis of the study

Figure 1 shows the enrolment process. The MIYI program was offered to all FEP and UHR youth (16–25 years) treated at BJCMHC. Inclusion criteria: (i) diagnosis of FEP, defined as Schizophreniform Disorder, Schizophrenia or Bipolar Affective Disorder 1 (BPAD1) as per Diagnostic and Statistical Manual of Mental Disorders‐5 (American Psychiatric Association, 2013) OR (ii) diagnosis of UHR as defined by Comprehensive Assessment of at Risk Mental States (Yung et al., 2005). Exclusion criteria: (i) medically unfit to participate in physical exercise as determined by a treating clinician OR (ii) intellectual disability (IQ <70). Fifteen young people consented to participate and completed the pre‐intervention measures. Of these, 12 participated in the program.

### 2.3 Intervention

The program consisted of weekly one‐hour classes delivered in a group setting across 12 weeks. Fifty minutes of yoga was delivered each session by a trained yoga instructor followed by 10 minutes of mindfulness exercises delivered by a clinical psychologist experienced in FEP. Yoga is a mind–body practice that combines physical postures, breathing techniques, and meditation (Cramer et al., 2017). The yoga instructor guided by a clinical psychologist adapted the yoga classes to match the energy level, attention ability and mood state of the group members. This resulted in some natural variation in each class. The program utilized three separate mindfulness exercises: present moment and breath awareness, body scan and non‐judgmental awareness. Each mindfulness exercise was repeated for four sessions. Clinicians from the FEP team attended all yoga classes to provide one to one assistance should any young consumer experience distress. Participants were invited to complete all classes.

### 2.4 Outcome measures

Demographics and background factors: Information including age, country of birth, Aboriginal and/or Torres Strait Islander identity, duration of care with service provider and prior yoga and mindfulness exposure was collected at baseline. Baseline medication use and changes to medication during the intervention were recorded.

Acceptability: This was measured by uptake of intervention, attendance, and intervention satisfaction. Uptake was measured as the percentage of participants who completed preintervention measures and attended at least one yoga class. Attendance was measured by the mean number of classes attended, percentage of participants who were “minimal users” i.e. who completed ≤3 yoga classes and reasons for non‐attendance. Intervention satisfaction was measured using a 16‐point questionnaire designed to evaluate participants' satisfaction with the intervention, perceived impact on their symptoms and likelihood of completing such an intervention again in the future. The questionnaire was administered by a member of the research team at the completion of the 12‐week intervention.

Safety: This was measured by the incidence of physical injury and by the participant's level of comfort, distress, and anxiety during the class. Physical injury was monitored during each class and a record log was kept of all incidents. Comfort, distress, and anxiety levels were measured by a six‐point questionnaire designed to evaluate whether participants felt unsafe, uncomfortable, distressed or anxious at any point during the sessions. The questionnaire was administered by a member of the research team at the completion of the 12‐week intervention.

Mental health outcomes including positive and negative psychotic, depression, anxiety and stress symptoms, sleep quality and functioning were measured pre‐ and postintervention to assess for deterioration in symptoms using clinically validated measures.

Positive and Negative Symptom Scale (PANSS): A clinician‐rated scale that provides an assessment of a participant's positive, negative and general psychopathology.

Global Assessment of Functioning Scale (GAF): A single‐item clinician‐rated scale that provides a global assessment of a participant's psychological, social and occupational functioning on a hypothetical continuum of mental health‐illness.

Depression, Anxiety and Stress Scale 21 (DASS21): A self‐report, 21‐item scale that measures symptoms of depression, anxiety and stress.

Pittsburgh Sleep Quality Index (PSQI): A self‐report instrument used to measure the quality and patterns of sleep in adults.

### 2.5 Analyses

Analyses were conducted using IBM SPSS Statistics v 25.0. For normally distributed variables, means, standard deviations (SD) and ranges were presented, otherwise medians and ranges were reported. Paired‐sample t‐tests were used to compare baseline and post‐ intervention scores for the continuous mental health variables. Mean differences (MD) and 95% confidence intervals (CI) were reported. Pearson correlations were used to determine if there was an association between baseline symptoms and attendance. Content analysis was used to analyse qualitative data.

## 3 RESULTS

### 3.1 Participants

Acceptability and safety components of the study were completed by all participants. Nine participants completed pre‐intervention and post‐intervention self‐reported measures. One participant was excluded from self‐report measures due to their inability to comprehend the questions and two participants did not complete self‐report measures. There were 12 participants (seven males, five females) with a mean age of 20.9 years (SD: 2.4, range 16.0–25.0) (Table 1). Ten (83.3%) were born in Australia and two (16.7%) overseas. No participants identified as Aboriginal and/or Torres Strait Islander. On average, participants had engaged with the service for 17.5 months (SD: 11.8 range 2.4–41.5) at the completion of the intervention. The most common diagnoses were schizophreniform disorder (*n* = 6, 50.00%), and UHR (*n* = 3, 25.0%). One participant (8.3%) was medication naïve, six (50.0%) were prescribed either one mood stabilizer, anti‐depressant or antipsychotic, four (33.3%) were prescribed a combination of one anti‐psychotic and one mood stabilizer or one anti‐depressant and one (8.3%) was prescribed two anti‐psychotics and one anti‐depressant. No changes occurred to mood stabilizer, anti‐depressant or antipsychotic prescribing during the study period. At baseline, nine participants (75.0%) reported previous sporadic practice of yoga, two (16.7%) had never practiced yoga, and one (8.3%) reported previous regular practice of yoga. Eight participants (66.7%) reported previous sporadic engagement with mindfulness therapies, while the other four (33.3%) reported having never done so.

**TABLE 1:** Participant characteristics—baseline demographic, clinical diagnosis and medication use of study participants

	Mean (SD)	Median (range)
Age (years)	20.92 (2.44)	21.00 (16–25)
Duration of care with service (months)	17.48 (11.80)	17.33 (2.37–41.49)

	*n*	%
Male	7	58.30
Born in Australia	10	83.30
Aboriginal and/or Torres Strait Islander	0	0.00
Diagnoses		
Schizophreniform disorder	6	50.00
BPAD1^a^	2	16.70
Schizophrenia	1	8.30
UHR^b^	3	25.00
Mood stabilizer		
Lithium	2	16.70
Anti‐depressant		
Escitalopram	3	25.00
Fluvoxamine	2	16.70
Anti‐psychotic		
Risperidone only	2	16.70
Quetiapine only	1	8.30
Olanzapine	2	16.70
Aripiprazole	1	8.30
Amisulpride	2	16.70
Risperidone and quetiapine	1	8.30

^a^ Bipolar affective disorder type 1.

^b^ Ultra‐high risk for psychosis.

### 3.2 Acceptability

**FIGURE 2:**
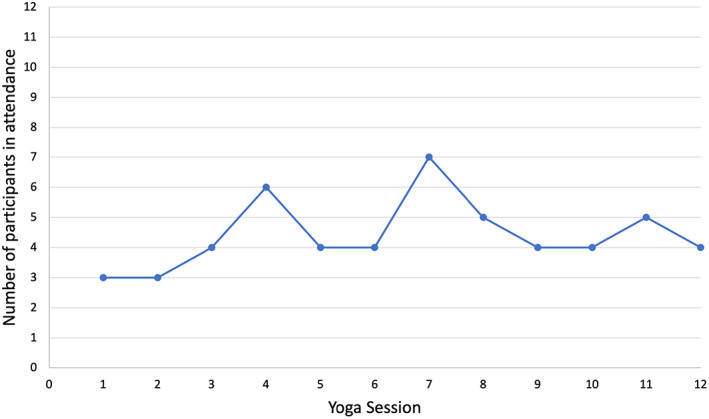
Graph of participant attendance at each intervention session—illustrates the number of participants who attended each intervention session

The initial uptake of MIYI was high: 12 out of 15 participants (80.0%) who completed baseline measures engaged with one or more yoga classes. Figure 2 illustrates the number of participants that attended each yoga session. On average, participants attended 4.4 (36.8%) yoga classes (SD: 3.0, range: 1.0–11.0). Five participants (41.7%) were deemed minimal users. On average, participants who were not minimal users (*n* = 7) attended 6.3 (52.5%) yoga classes (SD: 2.6, range: 4.0–11.0). On average, participants who were minimal users (*n* = 5) attended 1.8 (15.0%) yoga classes (SD: 0.8, range: 1.0–3.0). Participants' reasons for non‐attendance at any given session were recorded within 1 week of the session not attended. Reasons for non‐attendance varied: two (16.7%) did not attend due to study commitments, two (16.7%) due to employment commitments, two (16.7%) due to amotivation, two (16.7%) due to social engagements, one (8.30%) due to psychiatric symptoms, one (8.3%) due to a lack of interest in continuing with the program and two (16.7%) did not provide a reason. Nine participants (75.0%) reported being “very” to “extremely” satisfied with the yoga component and three (25.0%) reported being “slightly” to “moderately” satisfied. For the mindfulness component, nine participants (75.0%) reported being “very” to “extremely” satisfied, two participants (16.7%) reported being “slightly” to “moderately” satisfied and one participant (8.3%) reported being “unsatisfied”. One participant (8.3%) reported having difficulty understanding the yoga or mindfulness instructions.

Nine participants (75.0%) perceived the intervention to have improved their psychiatric symptoms, while the remainder (25.0%) perceived the intervention to have had no impact on these. Eleven participants (91.7%) also reported gaining some benefit from the intervention. Four participants (33.3%) reported learning relaxation techniques, three (25.0%) reported learning mindfulness techniques, three (25.0%) reported physical health benefits and two (16.7%) reported no benefit. Most participants expressed that they would continue to participate if the intervention were continued: 10 (83.3%) reported being “very” to “extremely” likely while two (16.7%) reported being “neutral” to continued participation.

### 3.3 Safety

There was no incidence of physical injury and all participants reported feeling safe during the yoga classes. Participants reported high levels of comfort during the classes: three (25.0%) reported being moderately comfortable, nine (75.0%) reported being very or extremely comfortable. Participants' levels of distress and anxiety during the yoga classes were minimal: nine (75.0%) reported none, three (25.0%) reported distress or anxiety in one class only. All participants (100.0%) who reported distress or anxiety in one class stated that it did not prevent them from attending future classes.

Participants exhibited mild positive (*M*: 15.5, range: 7.0–29.0, SD: 7.4), negative (*M*: 15.5, range: 7.0–25.0, SD: 5.2) and general (*M*: 33.8, range: 20.0–50.0, SD: 8.9) psychotic symptoms at pre‐intervention and mild positive (*M*: 11.3, range: 7.0–18.0, SD: 3.02), negative (*M*: 12.1, range: 7.0–20.0; SD: 4.4) and general (*M*: 28.1, range: 18.0–47.0, SD: 9.1) psychotic symptoms at post‐intervention. The change between pre‐ and postintervention scores for positive symptoms (MD: −4.3, 95% CI: −8.5 to 0.0), negative symptoms (MD: −3.4, 95% CI: −7.45 to 0.6) or general psychopathology (MD: −5.8, 95% CI: 11.8–0.2) was not statistically significant (all *p* > .05) as shown in Table 2. Participants had moderate functioning pre‐intervention (M: 54.5, range 40.0–75.0, SD: 12.1) and post‐intervention (M: 62.2, range 45.0–80.0, SD: 11.3). The improvement was statistically significant (MD: 7.7, 95% CI: 1.1–14.3) (*p* = .03) with a large effect size (*η* ^2^ = 0.66) as shown in Table 2.

**TABLE 2:** Pre‐intervention and post‐intervention mental health outcomes with mean changes. Comparison of changes in positive and negative psychotic symptoms, global functioning, depression, anxiety and stress symptoms and sleep quality

	*n*	Pre‐intervention			Post‐intervention			Mean change (95% CI)	*p*
		Mean	SD	Range	Mean	SD	Range		
Positive symptoms (PANSS‐P)^a^	12	15.50	7.39	7.00–29.00	11.25	3.02	7.00–18.00	−4.25 (−8.54 to 0.04)	.05
Negative symptoms (PANSS‐N)^b^	12	15.50	5.16	7.00–25.00	12.08	4.40	7.00–20.00	−3.42 (−7.45 to 0.62)	.10
General symptoms (PANSS‐G)^c^	12	33.83	8.93	20.00–50.00	28.08	9.09	18.00–47.00	−5.75 (−11.67 to 0.17)	.06
Functioning (GAF)^d^	12	54.50	12.14	40.00–75.00	62.17	11.30	45.00–80.00	7.67 (1.05–14.28)	.03*
Depression symptoms (DASS‐D)^e^	9	10.00	9.70	0.00–24.00	7.11	7.36	0.00–22.00	−2.89 (−7.94 to 2.16)	.22
Anxiety symptoms (DASS‐A)^f^	9	11.78	7.17	4.00–24.00	5.11	4.48	0.00–14.00	−6.67 (−10.27 to −3.06)	<.01*
Stress symptoms (DASS‐S)^g^	9	16.22	12.02	0.00–32.00	13.56	10.14	2.00–30.00	−2.67 (−9.41 to 4.08)	.40
Sleep quality (PSQI)^h^	9	7.67	4.74	2.00–15.00	6.00	3.12	2.00–11.00	−1.67 (−4.33 to 1.00)	.19

^a^ Positive and negative symptom scale‐positive symptoms.

^b^ Positive and negative symptom scale‐negative symptoms.

^c^ Positive and negative symptom scale‐general symptoms.

^d^ Global assessment of functioning.

^e^ Depression anxiety stress scales‐depression symptoms.

^f^ Depression anxiety stress scales‐anxiety symptoms.

^g^ Depression anxiety stress scales‐stress symptoms.

^h^ Pittsburgh sleep quality index.

*p < .05.

Participants reported mild depression (*M*: 10.0, range 0.0–24.0, SD: 9.7) and stress (*M*: 16.2, range 0.0–32.0, SD: 12.0) symptoms and moderate anxiety (*M*: 11.8, range 4.0–24.0, SD: 7.2) symptoms at baseline. Participants reported normal depression (*M*: 7.1, range 0.0–22.0, SD: 7.4), anxiety (*M*: 5.11, range 0.0–14.0, SD: 4.5) and stress (*M*: 13.6, range 2.0–30.0, SD: 10.1) symptoms at post‐intervention. The change in depressive (MD: ‐2.9, 95% CI: −7.9‐2.2) or stress symptoms (MD: −2.7, 95% CI: −9.4 to 4.1) was not statistically significant (all *p* > 0.05). The reduction in anxiety symptoms (MD: −6.7, 95% CI: −10.3 to −3.1) was statistically significant (*p* < .01) with a large effect size (*η* ^2^ = 1.0) as shown in Table 2.

Participants reported poor sleep quality at pre‐intervention (*M*: 7.7, range 2.0–15.0, SD: 4.7) and post‐intervention (*M*: 6.0, range 2.0–11.0, SD: 3.1). The mean improvement in sleep quality (MD: −1.67, 95% CI: −4.33 to 1.00) was not statistically significant (*p* > .05) as shown in Table 2. There was no association between pre‐intervention positive and negative psychotic symptoms and attendance (PANSS; *r* = −0.3, *p* = .3) or pre‐intervention depression, anxiety and stress symptoms and attendance (DASS21; *r* = −0.10, *p* = .8).

## 4 DISCUSSION

To our knowledge, this pilot study is the first to evaluate the acceptability and safety of a MIYI for young people with FEP or UHR in Australia. Consistent with our hypothesis, the yoga intervention was found to be an acceptable, well‐tolerated and safe intervention for delivery among the target sample. Initial uptake and interest in the MIYI were high, indicating that young service users are likely to be open to participating in nonpharmacological interventions. The overall attendance rate was somewhat lower than observed in previous studies undertaken in similar community settings (36.8% vs. 47.0%; Bhatia et al. (2017); Lin et al. (2015)). Figure 2 also illustrated that non‐attendance was spread across the 12‐week intervention. It appears sustaining attendance is a challenge for many studies in this population group. Young people reported a range of logistical and practical barriers to attendance (e.g. employment and study commitments) suggesting these interventions need to operate on a flexible and increasingly available timetable. Importantly, in the current study there was no relationship between attendance and mental health symptoms despite some participants reporting that amotivation and their mental health affected their engagement. Our finding differs from Lin et al. (2015) which found that female FEP participants who were more severely ill at baseline were more likely to be non‐adherent to the intervention. Satisfaction with MIYI was high with participants reporting a range of physical and mental benefits. A minority of participants reported having difficulty understanding the instructions. This suggests that this type of intervention may require additional modification to ensure that it is appropriate for the varying cognitive abilities of the sample. This may be achieved by involving the target sample in the design of the intervention and piloting aspects of the intervention before the study commences.

The intervention was shown to be safe with no incidence of physical injury and very low incidence of emotional distress. Importantly, there was no deterioration in any mental health symptom domain or function over the time course of the intervention. This is a major strength of the current study as past investigations have not reported on safety aspects of this new type of intervention (Cramer et al., 2013).

The results also provide initial support for the potential effectiveness of the MIYI in improving symptoms among FEP and UHR youth, however, caution must be taken when interpreting these findings due to study limitations. Participants reported lower levels of anxiety and higher levels of functioning at post‐intervention. Support for this also comes from studies in adults with schizophrenia (Behere et al., 2011). Additionally, Duraiswamy et al. (2007) found yoga was associated with improvement in positive and negative psychotic symptoms and Louise et al. (2018) found mindfulness interventions improved depression symptoms suggesting that similar interventions have additional benefits not observed in the current study.

### 4.1 Limitations

This study was limited by its small sample size, sample collection and non‐blinded and non‐controlled study design. A small convenience sample was used for this study. Participants who expressed interest in the intervention may have had increased engagement, compliance, and/or be more inclined to be satisfied with the intervention compared to individuals who did not express interest. The acceptability and safety questionnaires were completed at the completion of the 12‐week intervention meaning they relied on participants' accurate recall of satisfaction, comfort, distress, and anxiety. Future studies could assess participant's level of satisfaction, comfort, distress, and anxiety at the end of each session to more accurately capture this. Participants were largely stable in mental state and medication use at pre‐intervention. This along with duration of care with service indicates a high level of engagement with professional care. Whether these individuals are representative of all FEP or UHR youth clients, including those less engaged in community follow‐up, is unclear.

Participants and assessors were not blinded. The use of validated instruments to objectively record outcomes addressed this limitation to some extent. While the study found that MIYI improved anxiety symptoms and functioning in young people with FEP and UHR, the study did not employ a control population. It is possible that other factors will explain the changes in participants' symptoms and function observed.

Nonetheless, it is clear that the intervention was acceptable and did no harm to young consumers. Replication studies with larger sample sizes are needed to evaluate the clinical effectiveness of these novel interventions. Future studies would benefit from including a more diverse sample and actively targeting youth who are disengaged from traditional service models. They should also include a more rigorous study design including a standard care control and active comparator to account for possible confounding factors and blinding of assessors.

## CONFLICT OF INTEREST

The authors declare no conflicts of interest.

## Data Availability

The data that support the findings of this study are available on request from the corresponding author. The data are not publicly available due to privacy or ethical restrictions.
